# Supplementary material for: Pterosaur melanosomes support signalling functions for early feathers
Source: Nature. 2022 Apr 20;604(7907):684–8. doi: 10.1038/s41586-022-04622-3 (PMC9046085; doi:10.1038/s41586-022-04622-3)
Supplement: Supplementary file 1 — This file contains supporting text and supplementary references. [file 41586_2022_4622_MOESM1_ESM.pdf]

nature portfolio

https://doi.org/10.1038/s41586-022-04622-3

Supplementary information

Pterosaur melanosomes support signalling functions for early feathers

In the format provided by the authors and unedited

Supplementary information for:

Pterosaur melanosomes support signalling functions for early feathers

Aude Cincotta[1,2,3,4*], Michaël Nicolaï[5], Hebert Bruno Nascimento Campos[6], Maria McNamara[3,4*], Liliana d'Alba[5,7], Matthew Shawkey[5], Edio-Ernst Kischlat[8], Johan Yans[2], Robert Carleer[9], François Escuillié[10] & Pascal Godefroit[1]

[1]Directorate Earth and History of Life, Royal Belgian Institute of Natural Sciences, Brussels, Belgium. [2]Institute of Life, Earth and Environment, University of Namur, Namur, Belgium. [3]School of Biological, Earth and Environmental Sciences, University College Cork, Cork, Ireland. [4]Environmental Research Institute, University College Cork, Cork, Ireland. [5]Evolution and Optics of Nanostructures Group, Biology Department, Ghent University, Ghent, Belgium. [6]Centro Universitário Maurício de Nassau, Campina Grande, Paraíba, Brazil. [7]Naturalis Biodiversity Center, Leiden, The Netherlands. [8]Divisão de Bacias Sedimentares, Geological Survey of Brazil, Porto Alegre, Brazil. [9]Research Group of Analytical and Circular Chemistry, Institute for Material Research, Hasselt University, Diepenbeek, Belgium. [10]ELDONIA, Gannat, France.

Corresponding authors: aude.cincotta@ucc.ie, maria.mcnamara@ucc.ie

Contents

1. SUPPORTING TEXT. **3**

1.1. Origin of the specimen. **3**

1.2. Geological background. **3**

1.3. Description of the specimen. **3**

1.4. Soft tissue anatomy. **5**

SUPPLEMENTARY REFERENCES. **7**

1. Supporting text.

1.1. Origin of the specimen.

The specimen is believed to have been originally poached from an undetermined outcrop of the Early Cretaceous Crato Formation (Araripe Basin, north-eastern Brazil) and then resided in private collections in Europe for an unknown period of time. The specimen was deposited by its private owner at the Royal Belgian Institute of Natural Sciences (RBINS) through the French fossil preparation company Eldonia in 2020. The Brazilian authorities were contacted immediately and the specimen is now part of the collections of the Museum of Earth Sciences, Rio de Janeiro (collection number: MCT.R.1884). Negotiations between the RBINS, Eldonia and Brazilian authorities led to the official physical repatriation of the specimen to Brazil in early February 2022. The specimen was incompletely prepared when it arrived at Eldonia. Preliminary preparation under the supervision of FE allows us to certify that this specimen is not a composite and that restorations prior to its arrival at RBINS are limited to standard consolidation procedures.

1.2. Geological background.

The specimen is hosted within grey laminated limestones and was probably excavated from the Nova Olinda Member of the Crato Formation (Fm). This member is located at the base of the formation; all specimens of *Tupandactylus imperator* described thus far have been recovered from here[1–4]. The vertebrate assemblage in the Nova Olinda Member includes well preserved fish[5–8], bird feathers[9–15], lizards[16–19], anurans[20–23], turtles[24–28] and rare crocodilians[29–32]. The member also preserves abundant arthropods[33, 34], arachnids[35], crustaceans[36] and plants, including early angiosperms[37, 38].

The deposits of the Crato Fm consist of a mixture of siliciclastic deposits and laminated limestones[39]. The Nova Olinda Member is a laminated unit that for most of its vertical thickness (ca. 13 m) comprises pale carbonate-rich and dark carbonate-poor laminae[3]. This composition indicates deposition in a stratified water body characterized by a persistent thermocline or chemocline. The preservation of fossil soft tissues probably reflects (at least in part) low oxygen conditions in bottom waters. The geochemistry, sedimentology and palaeontology of the sediments indicate deposition in a brackish, restricted environment (not open marine) close to shore, probably a lagoon[39–40].

1.3. Description of the specimen.

Systematic Palaeontology.

Pterosauria Kaup, 1834

Pterodactyloidea Plieninger, 1901

Tapejaridae Kellner, 1989

Tapejarinae Kellner, 1989

Tupandactylus Kellner & Campos, 2007

Tupandactylus cf. *imperator* (Campos & Kellner, 1997)

Description and identification.

Specimen MCT.R.1884 comprises the posterior portion of the cranium and the remains of a soft tissue cranial crest, spanning five separate slabs. The specimen is in left lateral aspect, with most bones compacted. The absence of the anterior portion of the cranium is unlikely to represent an artefact of preservation. Instead, it was probably lost during excavation by quarry workers, as indicated by clean cuts (presumably saw cuts) through the bones in the anterior part of the cranium and by no obvious disarticulation around articulation sutures, which would be expected if the cranium disarticulated naturally from the rest of the carcass.

Of the cranium, parts of the orbit and nasoantorbital fenestra are still visible (Extended Data Figs. 1, 4a). Although only the dorsal part is preserved, the orbit appears rather small with an inverted pear geometry. The dorsal margin of the nasoantorbital fenestra is set more dorsally than the dorsal margin of the orbit. Orbit and nasoantorbital fenestra are separated by a thin lacrimal process. Two long bony processes extend posteriorly, supporting a soft tissue cranial crest that spans ca. 0.3 m^2 (465 mm high x 588 mm long). The occipital process is robust and extends posterodorsally from the back of the skull. Its ventral margin is straight, forming an angle of ca. 130° with the dorsal margin of the nasoantorbital fenestra; its dorsal margin is markedly concave. Only the posterior portion of the premaxillary bony crest is preserved as small rods, which seem to form a transition between the ossified base of the premaxillary crest and the soft tissue crest. These bony rods have been reported in the holotypes of *T. imperator*[41] and *T. navigans*[42]. The premaxillary bony crest extends as a postpremaxillary process that is about 20% longer (but more slender) than the parietal crest and is poorly preserved as a trabecular meshwork. The postpremaxillary process forms an angle of ca. 80° with the dorsal margin of the nasoantorbital fenestra and its posterior part has a distinct ventral arch.

The soft tissue cranial crest is the most complete known for *Tupandactylus*. The crest of MCT.R.1884 is similar to another described specimen of *Tupandactylus*[4], with a convex dorsoposterior margin. It comprises parallel fibres that run oblique to the occipital crest between the dorsal margin of the cranium and occipital crest. These parallel fibres are similar to the fibres described in other *Tupandactylus* specimens[1, 4]. Along the occipital process, the crest locally shows monofilaments and branched

integumentary structures, here identified as feathers (see Main Text). Similar monofilaments are known from the mandibular ramus of *Tupandactylus*[4] (reported as 'probable pycnofibres'), but have not been reported in association with a cranial crest previously. Overall, the cranial crest of this specimen closely resembles that of the tapejarid pterodactyloid *Tupandactylus* from the Crato Fm of the Araripe Basin[1]. Synapomorphic characters include the presence of a spine-like and slightly caudally-inclined postpremaxillary process and of a long (only slightly shorter than the postpremaxillary process) and caudally-oriented occipital crest[1, 4, 43]. As in previous specimens of *Tupandactylus*[1, 4, 42], no obvious sutures are present, indicating that the specimen was probably an adult.

The maximum extension of the occipital process of MCT.R.1884 cannot be inferred from the known specimens of *Tapejara wellnhoferi*[44], from the Santana Fm of the Araripe Basin, but it was obviously much shorter than in *T. imperator*. Further, our specimen differs from *T. wellnhoferi* whereby the orbit of the latter lies slightly below the dorsal margin of the nasoantorbital fenestra[43]. The occipital process of the tapejarid *Sinopterus dongi* differs from that of *T. imperator* in morphology and size[4, 45]. *Tupandactylus navigans*, also from the Crato Formation, lacks an occipital process and has a more vertically-inclined postpremaxillary process[42]. The specimen described in the present paper differs from the four other *T. imperator* specimens described from the Crato Formation whereby its occipital crest is more dorsally inclined and by the concave dorsal margin of the occipital crest[4]; the soft-tissue supracranial crest is narrower antero-posteriorly than that of the other *T. imperator* specimens. Such morphological variation is not unexpected and is implied in sexual selection[46]. We therefore tentatively refer this specimen to *Tupandactylus* cf. *imperator*, pending further evidence on the intraspecific variability of the cranial crest in this taxon.

1.4. Soft tissue anatomy.

In addition to the monofilaments and branched feathers associated with the occipital process (and that are described in detail in the main text), the cranial crest exhibits two types of fibrous integumentary structures. First, numerous sub-parallel, light to dark brown fibres (100–150 µm wide and up to ca. 300 mm long (Fig. 1a, Extended data Figs. 1, 4)) are widespread across the cranial crest. Those elongate fibres are well defined in the posteroventral part of the crest. They are faint or not evident in the anterodorsal part of the crest, where only a thick, black layer, possibly representing decayed organic matter, is present. Most of these fibres are mutually parallel, aligned with the sagittal axis ventrally and curved dorsally. Ventrally, a series of ca. 100 striking dark brown structures, each 600–900 µm wide, are aligned close to, but isolated from, the occipital process by a 20 mm to 30 mm-wide zone that lacks soft tissues (save faint fibre impressions). Each dark brown structure gives rise to a series of these sub-parallel dark brown fibres

(usually ca. five) (Extended Data Fig. 4a–c). The crest of *T. imperator* described in Pinheiro et al. (2011) also exhibits similar fibres, but those in MCT.R.1884 show a striking curvature.

Locally, the dorsal part of the crest displays wrinkles. Those are thin (ca. 0.3 mm wide), mutually parallel and tightly packed. In the posterior part of the crest, those structures are preserved as external moulds (Extended Data Fig. 4e, f) and run perpendicular to the main set of brown fibres that emerge from the ventral part of the crest. Those wrinkles are especially visible in the posterodorsal part of the crest (Extended Data Fig. 4e, f).

Supplementary references.

[1]Campos, D. A. & Kellner, A. W. A. Short note on the first occurrence of Tapejaridae in the Crato Member (Aptian), Santana Formation, Araripe Basin, Northeast Brazil. *Anais-Academia Brasileira de Ciencias* **69**, 83–88 (1997).

[2]Unwin, D. M. & Martill, D. M. Pterosaurs of the Crato Formation. In *The Crato Fossil Beds of Brazil: Window into an Ancient World* (eds Martill, D. M., Bechly, G. & Loveridge, R. F.), 475–524 (Cambridge University Press, Cambridge, 2007).

[3]Martill, D. M. & Frey, E. A new pterosaur Lagerstätte in NE Brazil (Crato Formation; Aptian, Lower Cretaceous): preliminary observations. *Oryctos* **1**, 79–85 (1998).

[4]Pinheiro, F. L., Fortier, D. C., Schultz, C. L., De Andrade, J. A. F. & Bantim, R. A. New information on the pterosaur *Tupandactylus imperator*, with comments on the relationships of Tapejaridae. *Acta Palaeontologica Polonica* **56**, 567–580 (2011).

[5]Brito, P. M., Martill, D. M. & Wenz, S. A semionotid fish from the Crato Formation (Aptian, Lower Cretaceous) of Brazil: palaeoecological implications. *Oryctos* **1**, e42 (1998).

[6]Davis, S. P. & Martill, D. M. The gonorynchiform fish *Dastilbe* from the Lower Cretaceous of Brazil. *Palaeontology* **42**, 715–740 (1999).

[7]Brito, P. M. The Crato Formation fish fauna. In *The Crato Fossil Beds of Brazil: Window into an Ancient World* (eds Martill, D. M., Bechly, G. & Loveridge, R. F.), 429–443 (Cambridge University Press, Cambridge, 2007).

[8]Brito, P. M., Yabumoto, Y. & Grande, L. New amiid fish (Halecomorphi: Amiiformes) from the Lower Cretaceous Crato Formation, Araripe Basin, northeast Brazil. *Journal of Vertebrate Paleontology* ***28***, 1007–1014 (2008).

[9]Kellner, A. A., Maisey, J. G. & Campos, D. A. Fossil down feather from the Lower Cretaceous of Brazil. *Palaeontology* **37**, 489–492 (1994).

[10]Martill, D. M. & Filgueira, J. M. A new feather from the Lower Cretaceous of Brazil. *Palaeontology* **37**, 483–487 (1994).

[11]Martill, D. M. & Frey, E. Colour patterning preserved in Lower Cretaceous birds and insects: the Crato Formation of NE Brazil. *Neues Jahrbuch für Geologie und Paläontologie-Monatshefte* **2,** 118–128 (1995).

[12]Martill, D. M. & Davis, P. G. A feather with possible ectoparasite eggs from the Crato Formation (Lower Cretaceous, Aptian) of Brazil. *Neues Jahrbuch für Geologie und Paläontologie-Abhandlungen* **219,** 241–259 (2001).

[13]Kellner, A. A. A review of avian Mesozoic fossil feathers. In *Mesozoic Birds: Above the Heads of Dinosaurs* (eds Chiappe, L. M. & Witmer, L. M.), 389–404 (University of California Press, Berkeley, 2002).

[14]Sayão, J. M., Saraiva, A. A. & Uejima, A. M. New evidence of feathers in the Crato Formation supporting a reappraisal on the presence of Aves. *Anais da Academia Brasileira de Ciências* **83**, 197–210 (2011).

[15]Prado, G. M., Anelli, L. E., Petri, S. & Romero, G. R. New occurrences of fossilized feathers: systematics and taphonomy of the Santana Formation of the Araripe Basin (Cretaceous), NE, Brazil. *PeerJ* **4**, e1916 (2016).

[16]Bonfim-Júnior, F. C. & Marques, R. B. Um novo lagarto do Cretáceo do Brasil. *Anuário do Instituto de Geociências* **20**, 233–240 (1997).

[17]Evans, S. E. & Yabumoto, Y. A lizard from the Early Cretaceous Crato Formation, Araripe Basin, Brazil. *Neues Jahrbuch fur Geologie und Paläontologie Monatshefte*, 349–364 (1998).

[18]Simões, T. R. Redescription of *Tijubina pontei*, an Early Cretaceous lizard (Reptilia; Squamata) from the Crato Formation of Brazil. *Anais da Academia Brasileira de Ciências* **84**, 79–94 (2012).

[19]Simões, T. R., Caldwell, M. W. & Kellner, A. W. A new Early Cretaceous lizard species from Brazil, and the phylogenetic position of the oldest known South American squamates. *Journal of Systematic Palaeontology* **13**, 601–614 (2015).

[20]Leal, M. E. C. & Brito, P. M. Anura do Cretáceo Inferior da Bacia do Araripe, Nordeste do Brasil. In *Paleontologia de Vertebrados: grandes temas e contribuic¸ õescientíficas* (eds Gallo, V., Brito, P. M., Silva, H. M. A. & Figueiredo, F. J.), 145–152 (Intercîencia, Rio de Janero, 2006).

[21]Báez, A. M., Moura, G. J. & Gómez, R. O. Anurans from the Lower Cretaceous Crato Formation of northeastern Brazil: implications for the early divergence of neobatrachians. *Cretaceous Research* **30**, 829–846 (2009).

[22]Carvalho, I. S., Agnolin, F., Rolando, M. A. A., Novas, F. E., Xavier-Neto, J., Freitas, F. I. & Andrade, J. A. F. G. A new genus of pipimorph frog (anura) from the early Cretaceous Crato formation (Aptian) and the evolution of South American tongueless frogs. *Journal of South American Earth Sciences* **92**, 222–233 (2019).

[23]Agnolin, F., Carvalho, I. S., Rolando, A. M. A., Novas, F. E., Xavier-Neto, J., Andrade, J. A. F. G. & Freitas, F. I. Early Cretaceous neobatrachian frog (Anura) from Brazil sheds light on the origin of modern anurans. *Journal of South American Earth Sciences* **101**, 102633 (2020).

[24]De Oliveira, G. R. & Kellner, A. W. A. Note on a plastron (Testudines, Pleurodira) from the Lower Cretaceous Crato Member, Santana Formation, Brazil. *Arquivos do Museu Nacional* **63**, 523–528 (2005).

[25]Fielding, S., Martill, D. M. & Naish, D. Solnhofen- style soft- tissue preservation in a new species of turtle from the Crato formation (Early Cretaceous, Aptian) of north- east Brazil. *Palaeontology* **48**, 1301–1310 (2005).

[26]Naish, D. Turtles of the Crato Formation. In *The Crato Fossil Beds of Brazil: Window into an Ancient World* (eds Martill, D. M., Bechly, G. & Loveridge, R. F.), 452–457 (Cambridge University Press, Cambridge, 2007).

[27]Romano, P. S., Oliveira, G. R., Azevedo, S. A., Kellner, A. W. & de Almeida Campos, D. New information about *Pelomedusoides* (Testudines: Pleurodira) from the Cretaceous of Brazil. *In* Brinkman,

D. B., Holroyd, P. A. & Gardner, J. D. (eds.), *Morphology and Evolution of Turtles*, 261–275. Springer, Dordrecht (2013).

[28]Oliveira, G. R. & Kellner, A. W. Rare hatchling specimens of *Araripemys* Price, 1973 (Testudines, Pelomedusoides, Araripemydidae) from the Crato Formation, Araripe Basin. *Journal of South American Earth Sciences* **79**, 137–142 (2017).

[29]Salisbury, S. W., Frey, E., Martill, D. M. & Buchy, M. C. A new crocodilian from the Lower Cretaceous Crato Formation of north-eastern Brazil. *Palaeontographica Abteilung A* **1–3,** 3–47 (2003).

[30]Frey, E. & Salisbury, S. W. Crocodilians of the Crato Formation: evidence for enigmatic species. In *The Crato Fossil Beds of Brazil: Window into an Ancient World* (eds Martill, D. M., Bechly, G. & Loveridge, R. F.), 463–474 (Cambridge University Press, Cambridge, 2007).

[31]Figueiredo, R. G. & Kellner, A. W. A new crocodylomorph specimen from the Araripe Basin (Crato Member, Santana Formation), northeastern Brazil. *Paläontologische Zeitschrift* **83**, 323 (2009).

[32]Figueiredo, R. G., Moreira, J. K., Saraiva, A. A. & Kellner, A. W. Description of a new specimen of *Susisuchus anatoceps* (Crocodylomorpha: Mesoeucrocodylia) from the Crato Formation (Santana Group) with comments on Neosuchia. *Zoological Journal of the Linnean Society* **163**, S273–S288 (2011).

[33]Menon, F. & Martill, D. Taphonomy and preservation of Crato Formation arthropods. In *The Crato Fossil Beds of Brazil: Window into an Ancient World* (eds Martill, D. M., Bechly, G. & Loveridge, R. F.), 79–96 (Cambridge University Press, Cambridge, 2007).

[34]Dias, J. J. & de Souza Carvalho, I. Remarkable fossil crickets preservation from Crato Formation (Aptian, Araripe Basin), a Lagerstätten from Brazil. *Journal of South American Earth Sciences* **98**, 102443 (2020).

[35]Dunlop, J. A., Menon, F. & Selden, P. A. Arachnida: spiders, scorpions and allies. In *The Crato Fossil Beds of Brazil: Window into an Ancient World* (eds Martill, D. M., Bechly, G. & Loveridge, R. F.), 103–132 (Cambridge University Press, Cambridge, 2007).

[36]Schweigert, G., Martill, D. & Williams, M., 2007. Crustacea of the Crato Formation. In *The Crato Fossil Beds of Brazil: Window into an Ancient World* (eds Martill, D. M., Bechly, G. & Loveridge, R. F.), 133–141 (Cambridge University Press, Cambridge, 2007).

[37]Mohr, B., de Oliveira, M. B. & Loveridge, B. The macrophyte flora of the Crato Formation. In *The Crato Fossil Beds of Brazil: Window into an Ancient World* (eds Martill, D. M., Bechly, G. & Loveridge, R. F.), 537–565 (Cambridge University Press, Cambridge, 2007).

[38]Lima, F. J., Saraiva, A. A., Silva, M. A. D., Bantim, R. A. & Sayao, J. M. A new angiosperm from the Crato Formation (Araripe Basin, Brazil) and comments on the Early Cretaceous Monocotyledons. *Anais da Academia Brasileira de Ciências* **86**, 1657-1672 (2014).

[39]Heimhofer, R. & Martill, D. The sedimentology and depositional environment of the Crato Formation. In *The Crato Fossil Beds of Brazil: Window into an Ancient World* (eds Martill, D. M., Bechly, G. & Loveridge, R. F.), 44–62 (Cambridge University Press, Cambridge, 2007).

[40]Martill, D. The geology of the Crato Formation. In *The Crato Fossil Beds of Brazil: Window into an Ancient World* (eds Martill, D. M., Bechly, G. & Loveridge, R. F.), 8–24 (Cambridge University Press, Cambridge, 2007).

[41]Kellner, A. W. A. New information on the Tapejaridae (Pterosauria, Pterodactyloidea) and discussion of the relationships of this clade. *Ameghiniana* **41**, 52–534 (2004).

[42]Frey, E., Martill, D. M. & Buchy, M. C. A new species of tapejarid pterosaur with soft-tissue head crest. *Geological Society, London, Special Publications* **217**, 65–72 (2003).

[43]Kellner, A. W. A. and Campos, D. D. A. Short note on the ingroup relationships of the Tapejaridae (Pterosauria, Pterodactyloidea). *Boletim do Museu Nacional* **75**, 1–14 (2007).

[44]Wellnhofer, P. The skull of Tapejara wellnhoferi Kellner (Reptilia, Pterosauria) from the Lower Cretaceous Santana Formation of the Araripe Basin, Northeastern Brazil. *Mitteilungen der Bayerischen Staatssammlung für Paläontologie und Historische Geologie* **31**, 89–106 (1991).

[45]Wang, X. & Zhou, Z. A new pterosaur (Pterodactyloidea, Tapejaridae) from the Early Cretaceous Jiufotang Formation of western Liaoning, China and its implications for biostratigraphy. *Chinese Science Bulletin* **48**, 16–23 (2003).

[46]Andersson, M. *Sexual Selection*, (Princeton University Press, Princeton, 1994).
