# Supplementary material for: Pterosaur melanosomes support signalling functions for early feathers
Source: Nature. 2022 Apr 20;604(7907):684–8. doi: 10.1038/s41586-022-04622-3 (PMC9046085; doi:10.1038/s41586-022-04622-3)
Supplement: Supplementary file 2 — Reporting Summary [file 41586_2022_4622_MOESM2_ESM.pdf]

nature portfolio

Corresponding author(s): Dr Aude Cincotta
Prof. Maria McNamara

Last updated by author(s): Feb 17, 2022

Reporting Summary

Nature Portfolio wishes to improve the reproducibility of the work that we publish. This form provides structure for consistency and transparency in reporting. For further information on Nature Portfolio policies, see our Editorial Policies and the Editorial Policy Checklist.

Statistics

For all statistical analyses, confirm that the following items are present in the figure legend, table legend, main text, or Methods section.

n/a	Confirmed	
☐	☒	The exact sample size (*n*) for each experimental group/condition, given as a discrete number and unit of measurement
☐	☒	A statement on whether measurements were taken from distinct samples or whether the same sample was measured repeatedly
☐	☒	The statistical test(s) used AND whether they are one- or two-sided<br>*Only common tests should be described solely by name; describe more complex techniques in the Methods section.*
☒	☐	A description of all covariates tested
☒	☐	A description of any assumptions or corrections, such as tests of normality and adjustment for multiple comparisons
☐	☒	A full description of the statistical parameters including central tendency (e.g. means) or other basic estimates (e.g. regression coefficient) AND variation (e.g. standard deviation) or associated estimates of uncertainty (e.g. confidence intervals)
☒	☐	For null hypothesis testing, the test statistic (e.g. *F*, *t*, *r*) with confidence intervals, effect sizes, degrees of freedom and *P* value noted<br>*Give P values as exact values whenever suitable.*
☒	☐	For Bayesian analysis, information on the choice of priors and Markov chain Monte Carlo settings
☒	☐	For hierarchical and complex designs, identification of the appropriate level for tests and full reporting of outcomes
☒	☐	Estimates of effect sizes (e.g. Cohen's *d*, Pearson's *r*), indicating how they were calculated

Our web collection on statistics for biologists contains articles on many of the points above.

Software and code

Policy information about availability of computer code

Data collection	No software was used to collect data
Data analysis	Melanosomes were measures using ImageJ freeware (version 64-bit Java 1.8.0_172; https://imagej.nih.gov/ij/); The significance of variation in the data was tested statistically using the ANOVA test of the freeware PAST (Palaeontological Statistics, version 4.09; https://www.nhm.uio.no/english/research/infrastructure/past/downloads/); Normality tests were done using RStudio freeware (version 1.1.463); Data on melanosome geometry was analysed using quadratic discriminant analysis (QDA) and multinomial logistic regression (MLR) using the MASS-package (Venables & Ripley, 2002) and the Nnet-package, both implemented in R using a published melanosome dataset (Babarović et al., 2019); Ancestral state estimations were performed using the methodology and data presented in Yang et al., (2019). We used maximum-likelihood estimations implemented in the 'ace' function of the ape 4 package (Paradis, 2011). Tree branch lengths were estimated using two methods: 'equal branch' length and 'minimum branch' length (mbl) using the 'DatePhylo' function in the strap R package (Bell & Loyd, 2015); The evolutionary models were run unsing the 'make.simmap' function of the phytools' package (Revell, 2012).

For manuscripts utilizing custom algorithms or software that are central to the research but not yet described in published literature, software must be made available to editors and reviewers. We strongly encourage code deposition in a community repository (e.g. GitHub). See the Nature Portfolio guidelines for submitting code & software for further information.

Data

Policy information about availability of data

All manuscripts must include a data availability statement. This statement should provide the following information, where applicable:

- Accession codes, unique identifiers, or web links for publicly available datasets
- A description of any restrictions on data availability
- For clinical datasets or third party data, please ensure that the statement adheres to our policy

Additional data, including dimension of melanosomes and the character matrix used in the phylogenetic analyses have been deposited in a data repository at Zenodo.org (DOI: 10.5281/zenodo.6122213). SEM images and samples are available from the corresponding authors on request.

Field-specific reporting

Please select the one below that is the best fit for your research. If you are not sure, read the appropriate sections before making your selection.

☐ Life sciences ☐ Behavioural & social sciences ☒ Ecological, evolutionary & environmental sciences

For a reference copy of the document with all sections, see nature.com/documents/nr-reporting-summary-flat.pdf

Ecological, evolutionary & environmental sciences study design

All studies must disclose on these points even when the disclosure is negative.

Study description	We report diverse melanosome geometries in the skin and simple and branched feathers associated with the cranial crest of a tapejarid pterosaur from the Early Cretaceous of Brazil (Crato Formation). We collected fossil soft tissue samples from the cranial crest itself and from both feather types. We imaged the samples using scanning electron microscopy (SEM) and measured the length and width of melanosomes from the SEM images. We imaged 22 samples in total.
Research sample	The research sample is a pterosaur cranial crest (Tupandactylus cf. imperator; MCT.R.1884). The specimen provides an almost complete cranial crest and two types of integumentary appendages. We targeted the soft tissue part of the crest (skin) and the feathers to study (1) their structure and (2) their taphonomy.
Sampling strategy	No statistical method was used to predetermine sample size. Small samples (a few mm wide) were collected in order to maintain the integrity of the fossil as much as possible. The size of the sample was sufficient to observe abundant melanosomes and have statistically significant data points.
Data collection	Twenty-two soft tissue samples were collected by A. Cincotta using sterile tools (tweezers). These samples are: (1) six independent monofilaments and branched feathers located around the posterior extension of the occipital process, (2) three fibres from the crest projecting from the base of the crest towards the occipital process, (3) four fibres collected on the posterior part of the crest, and (4) nine skin fibres located on the anterior part of the crest. Samples from the sedimentary matrix (from the region located between the base of the crest and the occipital process) were also collected. Samples were stored in SEM storage boxes before imaging.
Timing and spatial scale	Sample collection started in 2017 and finished in 2021. The timing for sample collection is related to the advance of our study. Timing of data collection has no importance in our study (fossil samples). Millimeter-sized samples were collected.
Data exclusions	No data were excluded from the analyses.
Reproducibility	Melanosome measurements, data for ancestral state estimations and other supplementary data are deposited in a data repository, available on:
Randomization	Samples were differentiated after their location on the fossil and their morphology: (1) cranial crest, (2) monofilaments and (3) branched feathers.
Blinding	Blinding is not relevant to our study because it does not involve randomised control trials.

Did the study involve field work? ☐ Yes ☒ No

Reporting for specific materials, systems and methods

We require information from authors about some types of materials, experimental systems and methods used in many studies. Here, indicate whether each material, system or method listed is relevant to your study. If you are not sure if a list item applies to your research, read the appropriate section before selecting a response.

Materials & experimental systems

n/a	Involved in the study	
☒	☐	Antibodies
☒	☐	Eukaryotic cell lines
☐	☒	Palaeontology and archaeology
☒	☐	Animals and other organisms
☒	☐	Human research participants
☒	☐	Clinical data
☒	☐	Dual use research of concern

Methods

n/a	Involved in the study	
☒	☐	ChIP-seq
☒	☐	Flow cytometry
☒	☐	MRI-based neuroimaging

Palaeontology and Archaeology

Specimen provenance	The fossil was originally collected from the Crato Formation at an unknown locality. The specimen resided in private collections for an unknown period of time before being deposited at the Royal Belgian Institute of Natural Sciences (RBINS). A cooperation agreement was signed on 11 October 2021 between RBINS and the embassy of Brazil in Belgium, which led to the official repatriation of the specimen to the Museum of Earth Sciences at Rio de Janeiro, Brazil, in early February 2022.
Specimen deposition	The specimen has been deposited at the Museum of Earth Sciences, Rio de Janeiro, Brazil. Collection number: MCT.R.1884.
Dating methods	No new dates are provided in our study.

☒ Tick this box to confirm that the raw and calibrated dates are available in the paper or in Supplementary Information.

Ethics oversight	No ethical approval was required as the specimen studied is a fossil. The specimen was repatriated to its country of origin as part of a joint collaboration between Brazil and Belgium, and in agreement with the 1972 UNESCO convention concerning the protection of the World cultural and natural heritage.

Note that full information on the approval of the study protocol must also be provided in the manuscript.
